# Enlarged, activated alveolar macrophages as quantitative surrogates of disease activity in pulmonary sarcoidosis

**DOI:** 10.3389/fmed.2026.1739663

**Published:** 2026-01-22

**Authors:** Manabu Ishida, Takeshi Saraya, Nobutaka Kitamura, Koh Nakata, Haruyuki Ishii

**Affiliations:** 1 Department of Respiratory Medicine, Kyorin University School of Medicine, Tokyo, Japan; 2 GM-CSF Inhalation Promoting Organization, Tokyo, Japan

**Keywords:** ACE, alveolar macrophages, cell enlargement, digital morphometry, rosette formation, sarcoidosis, vacuolation, soluble interleukin-2 receptor

## Abstract

**Background:**

In pulmonary sarcoidosis, alveolar macrophages (AMs) undergo epithelioid transformation, but their quantitative morphologic characteristics and association with systemic disease markers remain incompletely defined.

**Research question:**

Do enlargement and activation features of AMs in bronchoalveolar lavage (BAL) samples correlate with systemic markers of sarcoidosis activity (ACE and sIL-2R)?

**Methods:**

BAL cells from 16 biopsy-confirmed sarcoidosis cases and 4 healthy controls were cytocentrifuged, Diff-Quik®–stained, and analyzed using a digital planimetric microscope. Cell area (CA) of 50 randomly selected AMs per subject (total = 1,000) was quantified and categorized as small, medium, large, or extra-large based on control mean ± SD cutoffs. Nonparametric tests and principal component analysis (PCA) were applied to examine associations among CA, morphological features, serum ACE, and sIL-2R.

**Results:**

The mean CA was 31% greater in sarcoidosis than in controls (368.2 ± 169.3 μm^2^ vs. 281.4 ± 90.9 μm^2^; *p* < 0.001), with higher proportions of large/extra-large AMs (41% vs. 14%; *p* < 0.001). Vacuolation, rosette formation, and membrane ruffling were hallmarks of AM activation, correlating strongly with serum ACE and sIL-2R but not with the BALF CD4/CD8 ratio.

**Conclusion:**

AM enlargement and activation features are quantitative, reproducible surrogates of disease activity in pulmonary sarcoidosis. Clinical implications: Quantitative assessment of alveolar macrophage morphology may aid in assessment and monitoring of sarcoidosis activity and treatment response.

## Introduction

Sarcoidosis, first described by Besnier et al. in 1889, is a multisystem disease of unknown etiology characterized by non-caseating granulomatous infiltration of various tissues (1), most commonly the lungs. Although approximately two-thirds of patients experience spontaneous remission (55–90% in Stage I, 40–70% in Stage II, 10–20% in Stage III, and 0% in Stage IV), the remaining one-third progress to chronic disease with potentially life-threatening complications (2, 3). Granuloma formation is driven by interactions among alveolar macrophages (AMs), dendritic cells, and lymphocyte subsets, orchestrated by diverse cytokines and chemokines (4).

Histologically, multinucleated giant cells and AMs surrounded by epithelioid cells are typically located at the center of sarcoid granulomas. Ultrastructural studies suggest that sarcoid giant cells, rich in lysozyme-containing dense granules, arise from monocytes and AMs through cell fusion or proliferation (5, 6). In this context, accelerated granuloma formation reflects the activation of AMs and infiltration by monocytes differentiating into monocyte-derived macrophages, both of which are central to sarcoidosis pathogenesis and activity.

Transmission electron microscopy reveals that human AMs possess a complex ultrastructure, including an eccentrically placed kidney-shaped nucleus with peripheral chromatin, numerous surface folds, and abundant intracellular organelles such as lysosomes, mitochondria, and phagolysosomes. However, detailed morphological descriptions of AMs in sarcoidosis remain limited (7–10). The available reports describe highly irregular cell surfaces, marked cellular pleomorphism, electron-dense cytoplasmic inclusions (11), and other ultrastructural changes (7) consistent with macrophage activation. Morphometric analyses have reported AM diameters in sarcoidosis ranging from 14 μm (9) to 22 μm, (12) comparable to those in healthy subjects (mean 21.2 ± 0.3 μm) (13). However, several studies have identified a subset of sarcoidosis AMs with larger diameters than in healthy controls (7–9).

Hawley et al. (11) demonstrated that spontaneous macrophage–lymphocyte interactions were more prevalent in bronchoalveolar lavage fluid (BALF) from patients with sarcoidosis compared with healthy volunteers. Rosette formation, defined as more than one lymphocyte adhering to a single macrophage, was also observed more frequently in sarcoidosis (8, 11).

At the molecular level, tumor necrosis factor-*α* (TNF-α) induces intracellular adhesion molecule-1 (ICAM-1) expression on AMs, promoting cellular aggregation (14). Furthermore, leukocyte adhesion molecules (LeuCAMs) such as CD11a/b/c and CD18 are upregulated in sarcoid AMs relative to controls (15), contributing to lymphocyte recruitment to granulomas and the surrounding lung parenchyma.

According to the 1999 Joint Statement on Sarcoidosis from the American Thoracic Society, European Respiratory Society, and WASOG (3), “clinical activity” is defined as the onset, worsening, or persistence of symptoms or signs attributable to sarcoidosis. Assessment requires integration of clinical findings, biochemical and instrumental tests, and imaging. Markers of activity include elevated serum angiotensin-converting enzyme (ACE), hypercalcemia, declining lung function, lymphocytic alveolitis with a high CD4/CD8 ratio, and abnormalities on electrocardiogram, echocardiogram, thallium scan, or liver function tests. The 2020 ATS guideline (16) refined this definition, describing disease activity as “ongoing inflammation” reflected by new or worsening symptoms, loss of organ function, and progressive radiographic or other imaging changes.

In clinical practice, ACE (17, 18) and soluble interleukin-2 receptor (sIL-2R) (19, 20) are well-established markers of sarcoidosis activity.

We hypothesized that enlargement of AMs, accompanied by vacuolation and rosette formation, features well documented in sarcoidosis, correlates with disease activity as reflected by serum ACE and sIL-2R levels. To test this hypothesis, we quantitatively assessed AM cell areas using digital microscopy and compared the morphological characteristics of AMs between patients with sarcoidosis and healthy controls, considering both radiological stage and clinical activity.

## Methods

### Study design and participants

We conducted a retrospective study of 16 patients with histologically confirmed pulmonary sarcoidosis treated at Kyorin University Hospital between April 2014 and November 2015. Data from four healthy individuals described in our previous report (21), for whom written informed consent had already been obtained, served as controls. The study protocol was approved by the Institutional Review Committee of Kyorin University Hospital (Approval No. H23-085), which waived the requirement for new written informed consent through an opt-out procedure. All procedures adhered to relevant guidelines and regulations, including the Declaration of Helsinki.

### Cytocentrifuged bronchoalveolar lavage cells

BALF samples from patients and healthy controls were processed using an identical protocol. BAL was performed by instilling 50 mL of warmed saline three consecutive times, with each aliquot aspirated into a 50 mL syringe. The second and third aliquots were pooled for cell analysis. Within 1 h of collection, BALF was centrifuged at 500 × g for 5 min. The resulting cell pellet was resuspended in Hanks’ Balanced Salt Solution to a final cell density of 1 × 10^6^ cells/mL within 30 min. Aliquots (100 μL) were then loaded into a cytocentrifuge (Shandon Cytospin®) and spun at 500 rpm for 2 min. Slides were air-dried with a small fan for 30 min immediately after centrifugation. Differential cell counts were performed on 300 cells per slide following Diff-Quik® (modified Giemsa; Kokusai Shiyaku Co. Ltd., Kobe) staining according to the manufacturer’s protocol: 15 s in fixative solution, 10 s in Solution I, and 5 s in Solution II, followed by a gentle rinse with distilled water. Slides were then air-dried for another 30 min and coverslipped with Marinom™ mounting medium.

### Evaluation of cell area of AMs on digital microscope

We analyzed AMs using a digital microscope (BZ-X700; Keyence Corporation, Osaka, Japan, 2014) as previously described (22). The microscope’s built-in automated cell-counting software (Hybrid Cell Count Module, BZ-H4C; Keyence) was used to enumerate AMs and obtain preliminary measurements of CA and nuclear area (NA). Each AM was automatically assigned a unique sequential identifier, numbering cells from left to right across the screen. Contours of stained AMs were then manually adjusted. Morphometric measurements were performed by a single trained observer blinded to clinical data and biomarker levels. Automated cell detection was conducted using the microscope’s built-in software (Hybrid Cell Count Module, Keyence), which identified candidate cells and exported their area values. Because automated detection occasionally misidentified non–alveolar macrophages or cells with poor morphology, the observer performed standardized quality-control editing to refine cell contours and exclude ineligible cells (i.e., enucleated cells, cells with cytoplasmic disruption, cells lacking clearly discernible borders, or cells overlapping adjacent cells). The software’s fixed algorithm minimized operator-dependent bias, and manual editing was limited to removing misidentified or non-measurable cells. For each subject, 50 AMs were randomly selected from the eligible pool using Excel’s RAND function. Histograms of cell area were generated for each subject and for each group.

### Evaluation of morphological features

Morphological evaluation of each AM was performed using the following criteria: nuclear deviation (the nucleus is positioned at the periphery rather than centrally within the cytoplasm), nucleolar prominence (the nucleoli are clearly visible), homogeneous chromatin (the chromatin appears evenly stained), membrane ruffling (presence of microvilli or protrusions on the cell membrane), cytoplasmic vacuolation, and rosette formation (attachment of one or more lymphocytes to the plasma membrane of a single AM). AMs with cytoplasmic vacuoles occupying > 70% of the cytoplasm were operationally classified as exhibiting severe cytoplasmic vacuolation.

Multinucleated giant cells were defined as large, macrophage-like cells containing multiple (≥2) nuclei within a single cytoplasmic compartment.

### Correlation between morphological features and cell area

Principal component analysis (PCA) was used to explore relationships among cell-area variables, cellular morphology variables, clinical biomarkers, and other background factors, and to reduce dimensionality. The number of retained components was determined by eigenvalues >1 (Kaiser criterion), cumulative variance explained, and inspection of the scree plot. Promax rotation was applied to improve interpretability of the component loadings. A scatterplot of factor loadings for the first and second principal components was generated to visualize each variable’s contribution to the component structure.

### Statistical analysis

Nonparametric data were compared using the Mann–Whitney test, and categorical data were evaluated with the chi-squared test. All tests were two-sided, with *p* < 0.05 considered statistically significant. *p*-values were not adjusted for multiple comparisons and should be interpreted as reference values. Analyses were performed using SAS version 9.4 (SAS Institute Inc., Cary, NC), IBM SPSS Statistics version 29.0.1.0 (IBM, Armonk, NY), and R version 4.0.0 (The R Foundation for Statistical Computing).^1^

## Results

### Patients’ characteristics

Sixteen patients with sarcoidosis (7 males, 9 females; median age: 56 years, interquartile range [IQR]: 35–64) were included. Smoking status distribution was as follows: 44% former smokers, 25% current smokers, and 31% never-smokers. Chest X-ray staging revealed 3 patients at stage 0, 5 at stage I, 7 at stage II, and 1 at stage III. Median serum ACE and sIL-2R levels were 21.4 U/L (IQR: 17.4–34.4) and 763 U/mL (IQR: 370–1,095), respectively, with no significant differences across stages or smoking categories. BALF recovery yielded a median of 67.5% (IQR: 56.0–72.0) with a median total cell count of 1.0 × 10^5^ cells/mL (IQR: 0.7–1.8 × 10^5^). Differential cell counts demonstrated a predominance of AMs at 79.5% (IQR: 71.3–87.0) and lymphocytes at 19.5% (IQR: 12.3–28.8), with a median CD4/CD8 ratio of 4.1 (IQR: 2.8–7.0; Table 1). The control group consisted of four healthy male never-smokers (age range: 28–30 years). Their BALF contained a median of 93.2% AMs (IQR: 91.1–94.9) and 6.3% lymphocytes (IQR: 4.7–8.9).

**Table 1:** Characteristics of 16 patients with sarcoidosis.

Category	Sarcoidosis group (*N* = 16)
Gender (male/female)	7/9
Age, years (median [range])	56 (35–64)
Intercurrent Medical Illness (n)	
Respiratory diseases	0
Non-Respiratory diseases	4
Extrapulmonary lesion	
Uveitis	8
Uveitis/Skin	1
Cardiac	1
Liver/Spleen/Skin	1
Smoking status (ex/current/never)	7/4/5
Radiological stage (0/I/II/III/IV)	3/5/7/1/0
Serum marker	
ACE (U/L)	21.4 (17.4–34.4)
sIL-2R (U/mL)	763 (370–1,095)
BALF characteristics	
Percent recovery of retrieved fluid (%)	67.5 (56.0–72.0)
TCC (×10^5^/mL)	1.0 (0.7–1.8)
Macrophages (%)	79.5 (71.3–87.0)
Lymphocytes (%)	19.5 (12.3–28.8)
CD4/CD8 ratio	4.1 (2.8–7.0)

ACE, angiotensin-converting enzyme; BALF, bronchoalveolar lavage fluid; IQR, interquartile range; sIL-2R, soluble interleukin-2 receptor; TCC, total cell counts; CD4/CD8, CD4-positive to CD8-positive T-lymphocytes. All values are presented as median (interquartile range). Non-Respiratory diseases of four patients included old myocardial infarction, type 2 diabetes, chronic thyroiditis, and Guillain-Barré syndrome.

### Evaluation of cell area and nuclear area

**Figure 1:**
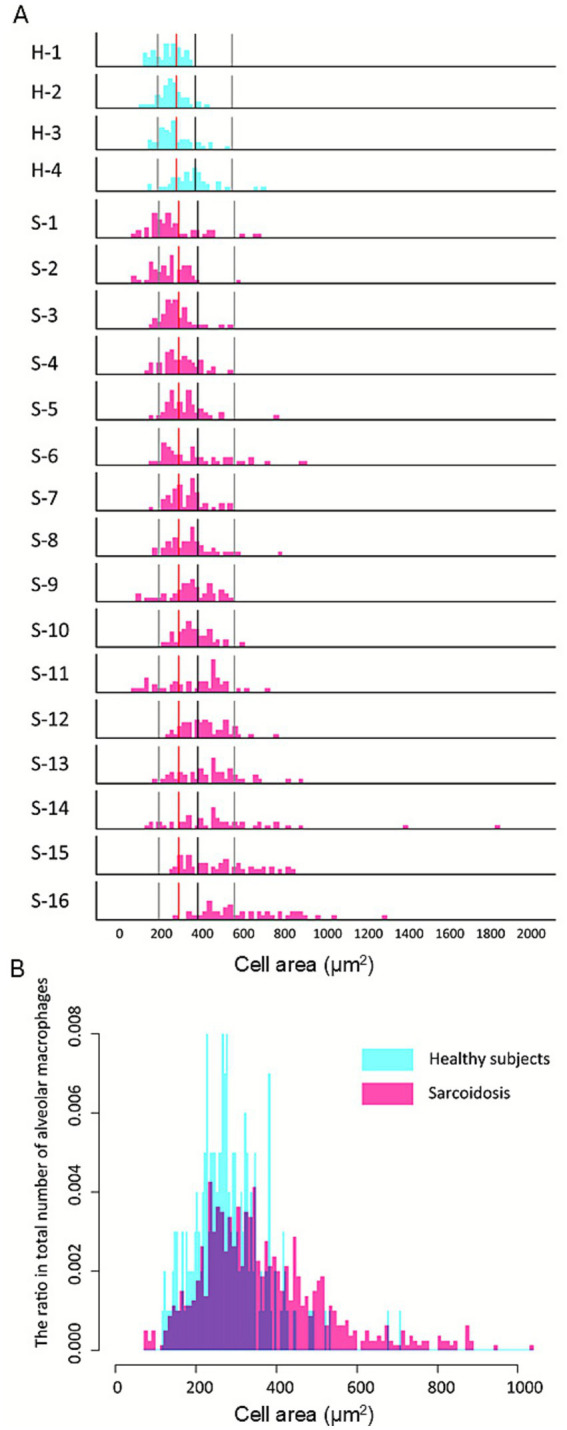
Increased proportion of large alveolar macrophages in pulmonary sarcoidosis. Histograms for each group **(A)** are arranged from top to bottom in ascending order of median cell area. Reference lines from left to right indicate the mean −1 SD, mean, mean +1 SD, and mean +3 SD of healthy subjects. Compared with healthy controls (H), the sarcoidosis group (S) demonstrates a broader distribution with a higher proportion of large and extra-large alveolar macrophages (AMs). Within the sarcoidosis group, the proportion of large/extra-large AMs exceeded 50% in S-11 (58.0%), S-12 (64.0%), S-13 (66.0%), S-14 (68.0%), S-15 (68.0%), and S-16 (90.0%), whereas all healthy cases remained below 50%: H-1 (40.0%), H-2 (12.0%), H-3 (6.0%), and H-4 (0%). The histogram illustrates the distribution of alveolar macrophage (AM) cell areas pooled from all subjects **(B)**. A total of 800 AMs were analyzed from sarcoidosis patients and 200 AMs from healthy controls. AMs from sarcoidosis patients (pink bars) show a broader distribution with a higher proportion of large and extra-large cells compared with healthy controls (blue bars), in whom small and medium-sized AMs predominate.

AMs from four healthy individuals were analyzed using the Hybrid Cell Count system, selecting 50 cells per subject. The mean and standard deviation (SD) were calculated from the total of 200 cells. Based on these values: small AMs were defined as those with an area < mean – SD (<190.5 μm^2^), medium AMs as those with an area between the mean - SD and mean + SD (190.5–372.3 μm^2^), large AMs as those with an area between the mean + SD and mean + 3SD (372.3–554.1 μm^2^), and extra-large AMs as those with an area > mean + 3SD (Supplementary Figure S1). The mean AM CA in sarcoidosis patients was 1.31-fold greater than in healthy controls (368.2 ± 169.3 μm^2^ vs. 281.4 ± 90.9 μm^2^; *p* < 0.001). Histograms for each case are shown in Figure 1A (H: healthy, *n* = 4) and (S: sarcoidosis, *n* = 16), arranged from top to bottom in ascending order of median CA. These demonstrate that the sarcoidosis group exhibited a broader CA distribution and higher proportion of large and extra-large AMs. Within the sarcoidosis group, the proportion of large/extra-large AMs ranged from 2.0 to 90.0%. Six cases—S-11 (58.0%), S-12 (64.0%), S-13 (66.0%), S-14 (68.0%), S-15 (68.0%), and S-16 (90.0%)—had proportions exceeding 50%. There was a strong positive correlation between the percentage of large/extra-large AMs and median CA (*r* = 0.97, *p* < 0.001). A combined histogram of 1,000 AMs from both groups confirmed that AMs from sarcoidosis patients (Figure 1B, pink columns) were predominantly distributed in the large and extra-large size categories compared with those from healthy subjects (Figure 1B, blue columns).

The proportions of small and medium-sized AMs were significantly higher in healthy subjects than in sarcoidosis patients (13.0% vs. 9.1%, *p* < 0.001; and 73.0% vs. 60.0%, *p* < 0.001, respectively; Table 2). Conversely, the proportions of large and extra-large AMs were significantly higher in sarcoidosis patients than in healthy subjects (28.6% vs. 12.5%, *p* < 0.001; and 12.3% vs. 1.5%, *p* < 0.001, respectively). Overall, AMs from sarcoidosis patients displayed a broad size spectrum, ranging from distributions similar to healthy controls to profiles dominated by large and extra-large cells.

**Table 2:** Distribution of alveolar macrophage (AM) area classified by mean and standard deviation in sarcoidosis patients and healthy controls (50 cells per subject).

AM area category	Healthy controls (n=4; 200 AMs)	Sarcoidosis patients (n=16; 800 AMs)	*p*-value
Small (<190.5 μm^2^)	26 (13.0%)	73 (9.1%)	<0.001
Medium (190.5–372.3 μm^2^)	146 (73.0%)	400 (50.0%)	<0.001
Large (372.3–554.1 μm^2^)	25 (12.5%)	229 (28.6%)	<0.001
Extra-large (> 554.1 μm^2^)	3 (1.5%)	98 (12.3%)	<0.001

Values are expressed as the number (%) of AMs out of the total counted (50 cells per case). Cell area was classified into small (< mean − 1.0 SD), medium (mean ± 1.0 SD), large (mean + 1.0 SD to mean + 3.0 SD), and extra-large (> mean + 3.0 SD) categories.

### Comparison of AMs in healthy participants and sarcoidosis with representative morphological features of sarcoidosis AMs on light microscope

**Figure 2:**
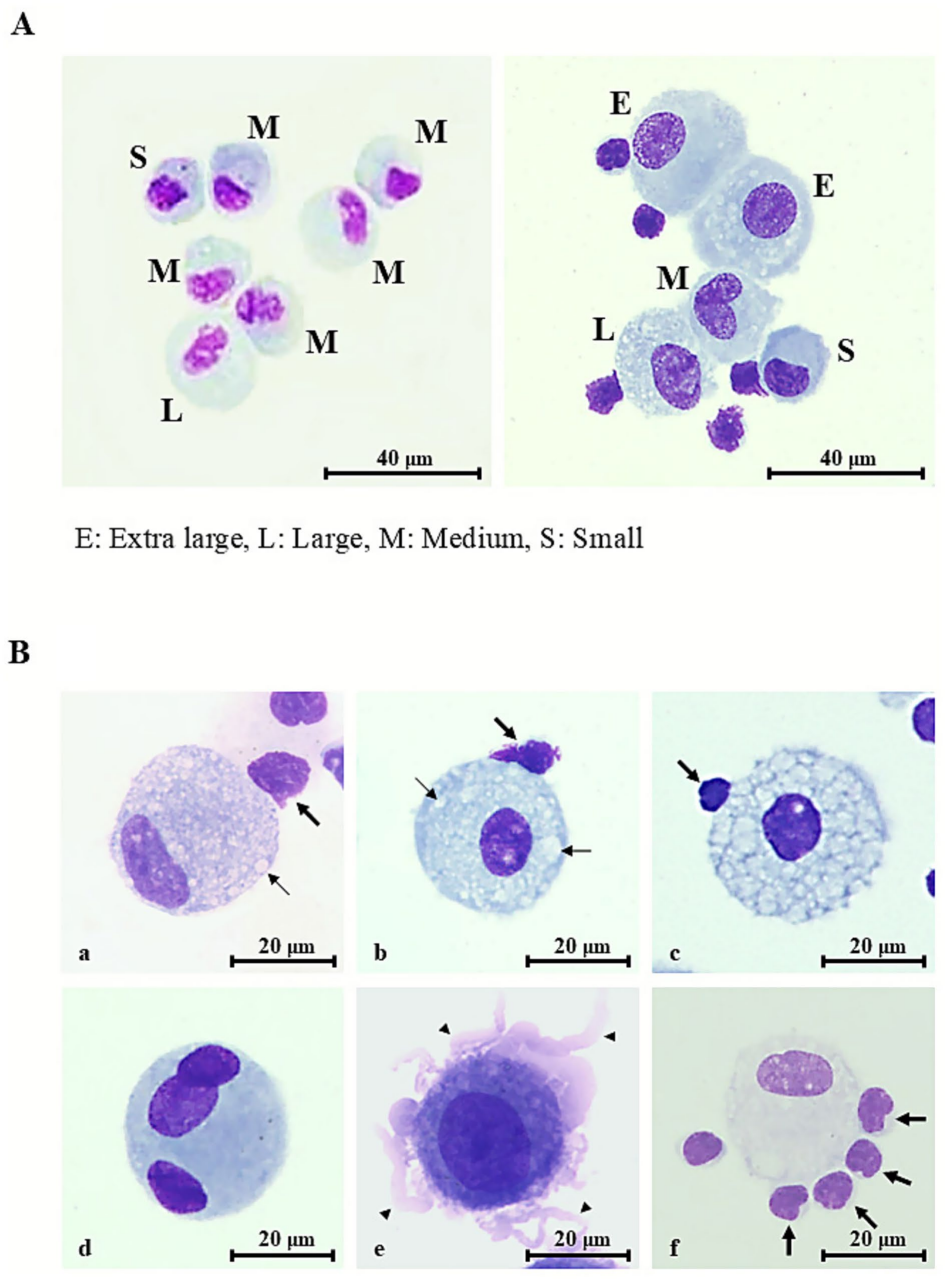
Representative morphologic features of alveolar macrophages in healthy controls and sarcoidosis. **(A)** Representative Diff-Quik**®** –stained images of alveolar macrophages (AMs) in bronchoalveolar lavage fluid specimens (scale bar = 40 μm, shown in figure). E, extra-large; L, large; M, medium; S, small. **(A, left panel)** Healthy individual: AM measuring 15–20 μm in diameter, with faintly basophilic cytoplasm and elongated, thin nuclei displaying unevenly distributed chromatin. Occasional cytoplasmic vacuoles are present. **(A, right panel)** Sarcoidosis patient: AMs enlarged to 20–30 μm with bright basophilic cytoplasm, round nuclei exhibiting relatively uniform chromatin, and prominent nucleoli. Numerous vacuolated AMs and lymphocyte attachment to the AM surface (rosette formation) are also observed. **(B)** Typical morphological features of sarcoidosis AMs (scale bar = 20 μm, shown in figure). **(a)** Mild-to-moderate cytoplasmic vacuolation (thin arrows) with nuclear deviation. **(b)** Mild-to-moderate vacuolation (thin arrows) without nuclear deviation. **(c)** Severe vacuolation, with vacuoles occupying more than 70% of the cytoplasm. **(d)** Multinucleated giant cell containing three oval-to-round nuclei with coarse chromatin and conspicuous nucleoli. **(e)** Membrane ruffling with numerous villi (thick arrows) protruding from the cell surface. **(f)** Rosette formation with numerous lymphocytes attached to the AM surface (thick arrows). Rosette formation is also observed in **(a–c)**.

Stained images of AMs from sarcoidosis patients (Figure 2A, right panel) were compared with those from healthy subjects (Figure 2A, left panel). In healthy subjects, AM cytoplasm stained faintly basophilic, whereas many AMs from sarcoidosis patients appeared deeply basophilic. Sarcoidosis AMs demonstrated homogeneous chromatin with prominent nucleoli (Figure 2A, right panel). Nuclei in sarcoidosis AMs were preferentially located at the cell periphery (Figures 2Ba), whereas in healthy AMs, nuclei were typically central or only slightly eccentric, indicating nuclear polarity in sarcoidosis (Figure 2A, right panel).

Cytoplasmic vacuolation ranged from mild to moderate (Figures 2Ba, Bb) to severe (Figures 2Bc), with vacuoles occupying nearly the entire cytoplasm. Mild-to-moderate and severe vacuolation were observed in 14.2 and 2.2% of sarcoidosis AMs, respectively.

Multinucleated giant cells were comparable in size to, or larger than, vacuolated sarcoidosis AMs. They exhibited faintly basophilic cytoplasm containing three nuclei (Figures 2Bd). Nuclei were oval to round with uniform to coarsely granular chromatin and often displayed prominent nucleoli (Figures 2Bd). These cells accounted for 5.7% of sarcoidosis AMs.

Pronounced membrane ruffling, characterized by broad, irregular lamellipodial protrusions and delicate frilled folds that produced a jagged, scalloped outline of the plasma membrane (Figures 2Be), was observed in 14.1% of sarcoidosis AMs.

Rosette formation was identified in 14.9% of sarcoidosis AMs, with up to five lymphocytes adhering to a single AM (Figures 2Bf), resembling the patterns seen in Figure 2A (right panel) and Figures 2Ba–c. Most rosettes consisted of a single lymphocyte (10.5%), while fewer comprised two (2.6%), three (1.2%), four (0.4%), or five (0.3%) lymphocytes. In healthy volunteers, rosette formation was identified in 12.8% of AMs, consisting of rosettes with one lymphocyte (10.7%) or two lymphocytes (2.1%); rosettes with three or more lymphocytes were not observed (0%). Collectively, sarcoidosis AMs exhibited a wide spectrum of morphological alterations consistent with cellular activation.

CA correlated positively with nuclear area (*r* = 0.600, *p* < 0.001), with a stronger association in vacuolated cells (*r* = 0.578, *p* < 0.001) than in non-vacuolated cells (*r* = 0.457, *p* < 0.001; Spearman’s rank correlation coefficient; Supplementary Figure S2), indicating that specific morphological traits contribute to AM enlargement.

### Correlation of cell area with morphological features and contributing factors to AM enlargement

**Figure 3:**
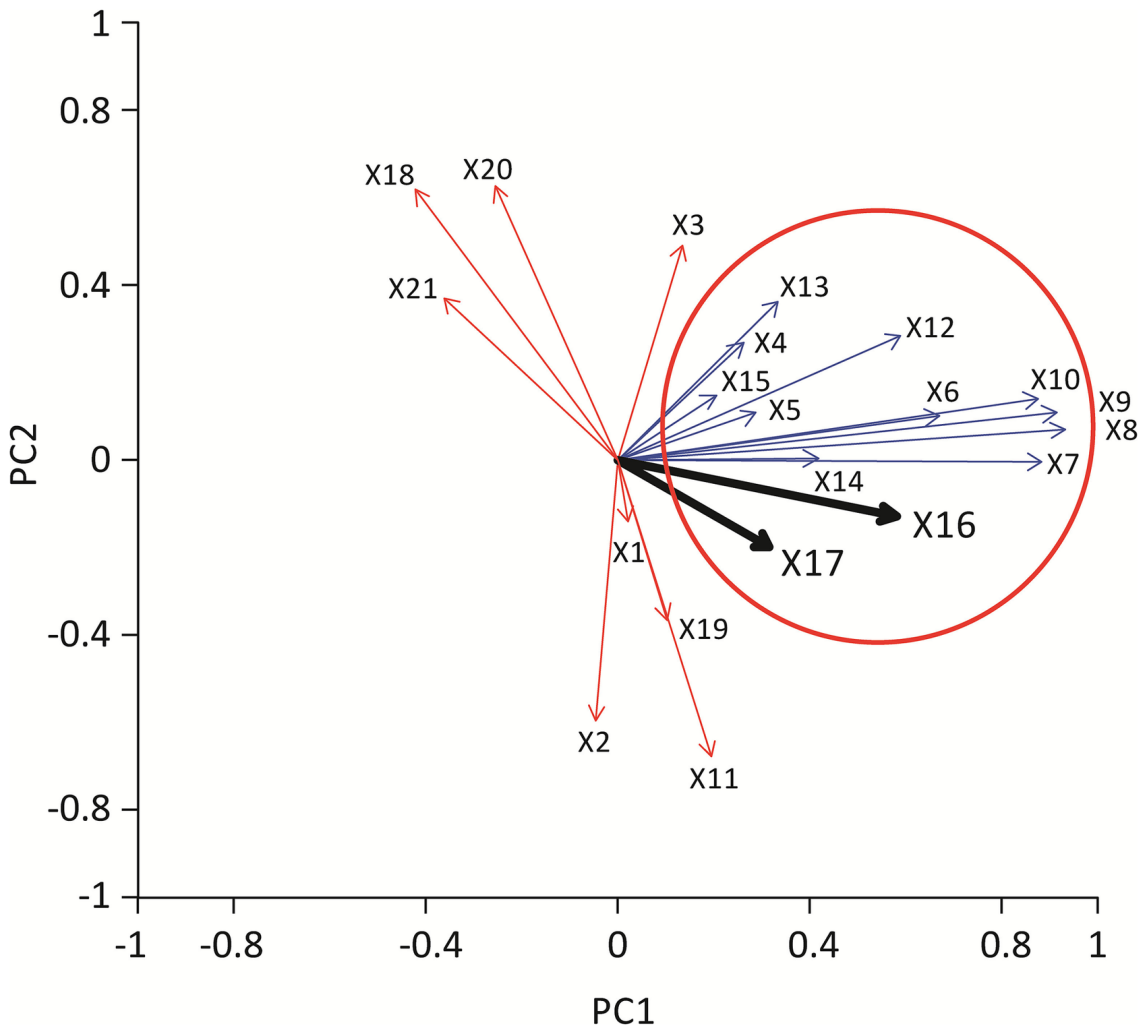
Vector diagram showing the results of principal component analysis. Each arrow represents a variable included in the analysis, with arrow length reflecting the contribution to the principal components. The thick black arrow indicates the vector for serum ACE level and serum sIL-2R level. Parameters pointing in a similar direction (blue arrows) demonstrate stronger positive correlations, whereas vectors pointing in opposite directions (red arrows) indicate inverse relationships. Together, the analysis illustrates that alveolar macrophage enlargement, cytoplasmic vacuolation, and rosette formation cluster closely with serum ACE and sIL-2R, suggesting that these morphological features correlate with markers of disease activity. X1 deviation of nucleus, X2 homogeneous chromatin, X3 nucleolar prominence, X4 cytoplasmic vacuolation, X5 ruffling, X6 rosette formation, X7 rosette 1, X8 rosette 2, X9 rosette 3, X10 rosette 4, X11 multinucleated giant cell, X12 cell area, X13 nuclear area, X14 N/C ratio, X15 lym, X16 ACE, X17 sIL2R, X18 age, X19 stage, X20 CD4/CD8 ratio, X21 gender.

To assess multiple variables simultaneously, including AM CA, morphological features (vacuolation, rosette formation, membrane ruffling), and clinical biomarkers (serum ACE, sIL-2R, CD4/CD8 ratio), we performed PCA (Figure 3). PCA is a statistical technique that reduces high-dimensional data into a few “principal components,” allowing visualization of relationships among variables. The first principal component (PC1) accounted for approximately 27% of the variance. CA, cytoplasmic vacuolation, rosette formation, membrane ruffling, and serum ACE and sIL-2R clustered together on the loading plot, suggesting covariation between enlarged, activated AMs and elevated ACE/sIL-2R levels. The second component (PC2) accounted for 13% of the variance and diverged from radiographic stage and BAL lymphocyte CD4/CD8 ratio vectors, suggesting weaker covariance with AM morphometric variables in this dataset.

These PCA findings are consistent with the interpretation that AM enlargement with vacuolation/rosette formation is associated with ACE and sIL-2R levels; however, PCA is exploratory and does not imply mechanistic relationships.

**Figure 4:**
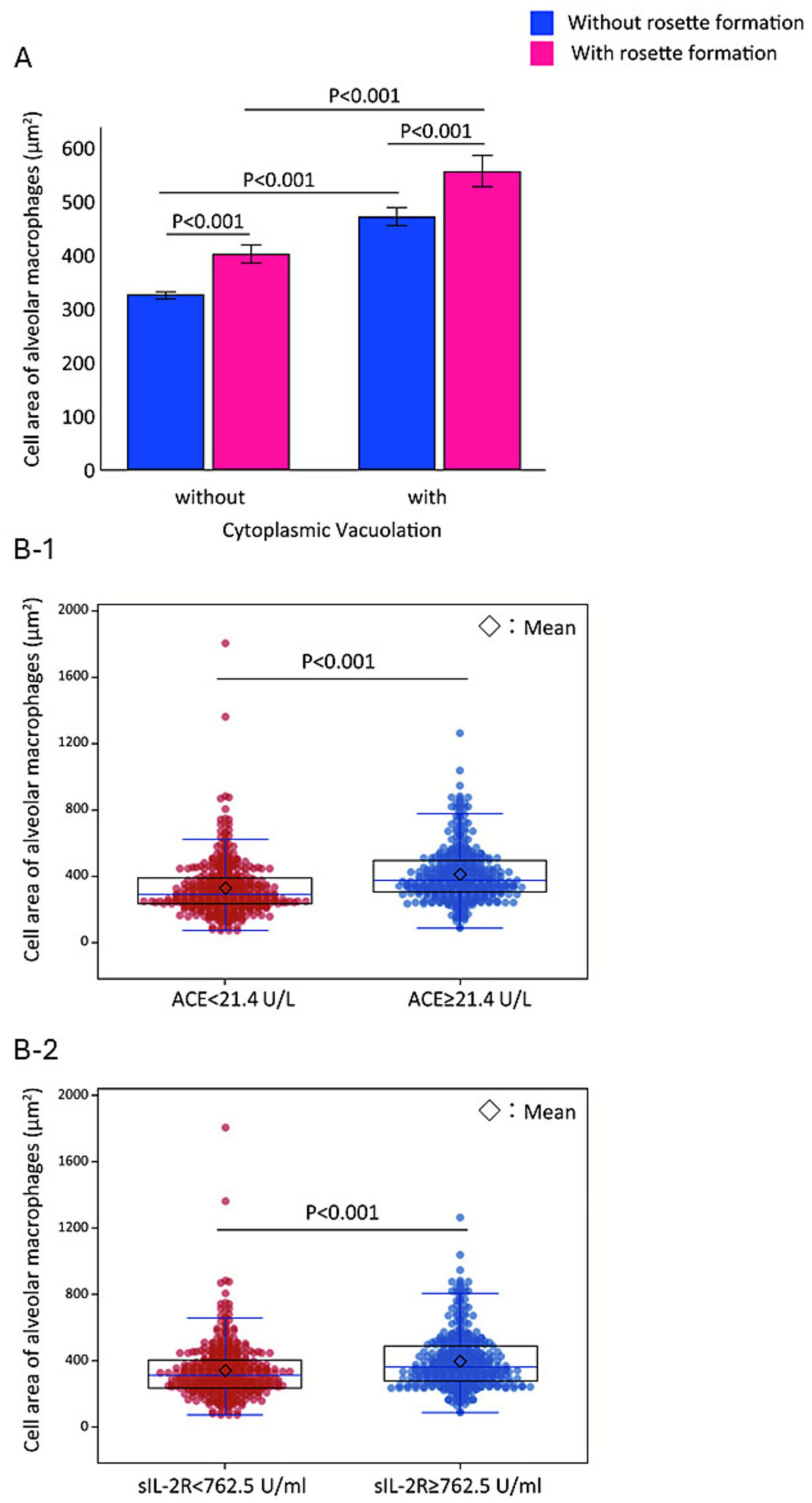
Stepwise enlargement of alveolar macrophages associated with vacuolation, rosette formation, and elevated serum markers. Fifty alveolar macrophages (AMs) are analyzed per case (*n* = 16) **(A)**. Vacuolation-positive AMs (mean ± SE, 500.3 ± 15.4 μm^2^) are significantly larger than vacuolation-negative AMs (337.1 ± 6.1 μm^2^, *p* < 0.001). Independently, AMs exhibiting rosette formation (474.7 ± 16.7 μm^2^, pink columns) have greater cell areas than those without rosette formation (349.4 ± 6.4 μm^2^, blue columns, *p* < 0.001). Mean ± SE cell area increases progressively from left to right across the four columns—319 ± 6.0 μm^2^, 423.5 ± 18.5 μm^2^, 469 ± 17.4 μm^2^, and 574 ± 29.5 μm^2^—demonstrating a stepwise enlargement of AMs. Bee swarm plots of AM cell area (*n* = 800) **(B)** demonstrate that patients with elevated ACE levels (≥21.4 U/L) have significantly larger AMs (median 375.5 μm^2^, IQR: 302.8–492.5) compared with those with lower ACE (< 21.4 U/L; median 290.5 μm^2^, IQR: 231.0–392.0; *p* < 0.001) **(B-1)**. Patients with elevated sIL-2R levels (≥762.5 U/mL) have significantly larger AMs (median 360.5 μm^2^, IQR: 280.0–490.5) compared with those with lower sIL-2R (< 762.5 U/mL; median 313.0 μm^2^, IQR: 237.0–405.8; *p* < 0.001) **(B-2)**.

To validate PCA findings, we performed pairwise analyses. Among 800 AMs (Figure 4A), those with rosette formation (red boxes) were significantly larger than those without (blue boxes) in both vacuolated and non-vacuolated groups (*p* < 0.001 for both). Rosettes involving two or more lymphocytes (≥2) were associated with further increases in AM size (Figure not shown). A beeswarm plot of AM CA (*n* = 800; Figure 4B) demonstrated that patients with elevated ACE (≥ 21.4 U/L) had significantly larger AMs than those with lower ACE (< 21.4 U/L, *p* < 0.001; Figures 4B1). Similarly, patients with elevated sIL-2R (≥ 762.5 U/mL) had significantly larger AMs than those with lower sIL-2R (< 762.5 U/mL, *p* < 0.001; Figures 4B2). ACE and sIL-2R levels correlated strongly with each other (*r* = 0.813, *p* < 0.001). These findings suggest that enlargement of AMs, mediated by cytoplasmic vacuolation and rosette formation, is tightly linked to the disease activity markers ACE and sIL-2R.

## Discussion

This study demonstrates that AMs derived from patients with sarcoidosis have a substantially larger CA—approximately 1.3-fold greater than that of healthy controls—when quantified using a computer-assisted planimetric method. Sarcoidosis AMs displayed considerable heterogeneity in cell size, with large and extra-large cells frequently exhibiting vacuolization and rosette formation. Notably, these enlarged AMs were associated with elevated serum ACE and sIL-2R levels. Although the morphological abnormalities of AMs in sarcoidosis have long been recognized by both pathologists and pulmonologists, no clear consensus has emerged. Our findings suggest that such morphological characteristics may serve as quantitative surrogate markers of disease activity. Serum ACE and sIL-2R are commonly used adjunctive biomarkers in sarcoidosis because they reflect granulomatous and immune activation. ACE is produced by activated monocytes/macrophages and epithelioid cells within granulomas, whereas sIL-2R reflects T-cell activation and has been associated with disease activity in clinical practice. Thus, elevations in these markers are generally interpreted as indicating a higher inflammatory/granulomatous burden (i.e., “activity” rather than irreversible fibrotic damage), which provides a plausible biological basis for their association with enlarged, activated AMs observed at BAL.

In this study, AMs were classified into four size categories based on CA measured by digital morphometry, corresponding approximately to the following diameters (calculated by assuming a circular rather than an oval shape): small (<15.6 μm), medium (15.6–21.8 μm), large (21.8–26.6 μm), and extra-large (>26.6 μm). Previous morphometric studies have reported that the diameter of healthy controls ranges from 12 μm (8, 9) to 20 μm (10, 21), values that largely correspond to the small or medium categories in our classification, even in sarcoidosis AMs (9, 12). In contrast, our analysis identified a predominance of large and extra-large AMs exceeding 20 μm in diameter in patients with sarcoidosis. The mechanisms underlying AM enlargement remain incompletely understood. Proposed explanations include accelerated protein synthesis (9), accumulation of dense cytoplasmic deposits (8), and increased numbers of small organelles, such as lysosomes, phagolysosomes, myelin-like structures, mitochondria, and Golgi apparatus, observed by electron microscopy. These ultrastructural features are consistent with heightened metabolic activity and cellular activation. Furthermore, aberrant mTOR signaling and non-JAK–STAT proliferative pathways—both implicated in sarcoidosis tissue growth (23, 24)—may also contribute to the emergence of these oversized AM subsets, which are significantly larger than those found in healthy individuals.

A comparable enlargement has also been observed in AMs from patients with chronic granulomatous disease (CGD). These AMs are significantly larger than those from non-CGD controls (10)—including individuals with feeding intolerance, gastroesophageal reflux, cough, obstructive sleep apnea, asthma, or pneumonia—and exhibit a foamy, vacuolated cytoplasm on light microscopy. Electron microscopy further reveals that their cytoplasm is densely packed with enlarged lysosomes containing lipofuscin-like or myelin-like residual bodies, corroborating their activated state. Because cytoplasmic vacuolation and a foamy appearance are non-specific features seen in multiple conditions—including hypersensitivity pneumonitis, drug-induced pneumonitis, toxic inhalation injury, and lysosomal storage diseases (10)—sarcoidosis should always be included in the differential diagnosis.

A spontaneous interaction between AMs and lymphocytes—referred to as rosette formation—was observed more frequently in patients with sarcoidosis than in healthy subjects (8). Notably, in sarcoidosis, individual AMs often carried more than two adherent lymphocytes (11). In the present study, both rosette formation and cytoplasmic vacuolation were independently associated with AM enlargement on two-factor analysis.

In an exploratory analysis, the BAL lymphocyte proportion correlated positively with rosette-related indices (Rosette1–Rosette4, defined by ≥1 to ≥4 adherent lymphocytes; Spearman’s *ρ* = 0.575–0.912, *p* = 0.0197 to 8.38 × 10^−7^), suggesting that AM–lymphocyte adhesion increases with the degree of lymphocytic alveolitis, consistent with the clustering observed in the PCA. This observation supports the biological plausibility of rosette formation as a morphologic readout of local immune activation in pulmonary sarcoidosis.

The underlying mechanisms of this cellular aggregation likely involve: over-expression of adhesion molecules on AMs, including ICAM-1 (CD54), CR3 (CD11b/CD18) (25–27), and LFA-1 (CD11a/CD18) (26); and cytokine-driven up-regulation of ICAM-1, with TNF-*α*- or IFN-*γ* promoting expression of ICAM-1 and stabilizing lymphocyte–macrophage attachment (4).

Prior studies have shown that AMs obtained from patients with clinically active sarcoidosis display higher expression of ICAM-1, CR3, and LFA-1 and produce more TNF-α than AMs from patients with inactive disease or from healthy subjects.

Activated AMs therefore orchestrate the initial accumulation, aggregation, and fusion of the cellular building blocks required for granuloma formation, driven chiefly by TNF-α and reinforced by NK cell–derived IFN-γ (4). Pueringer et al. (28) reported that sarcoidosis AMs spontaneously secrete TNF-α, IL-1β, and prostaglandin E₂, with further augmentation after *in vitro* lipopolysaccharide stimulation. Similarly, Ziegenhagen et al. (29) demonstrated elevated spontaneous TNF-α release and showed that patients with high TNF-α release or elevated serum sIL-2R at diagnosis had a significantly greater risk of disease progression even without initial steroid therapy. Pforte et al. reported that the proportion of IL-2R-positive AMs (25.0%) was 3.5-fold higher than that of alveolar lymphocytes (30). Both IL-2R expression on AMs and circulating sIL-2R levels decreased with immunosuppressive therapy, whereas IL-2R expression on alveolar lymphocytes remained unchanged. Collectively, these findings underscore the central role of activated AMs in the pathogenesis and progression of pulmonary sarcoidosis.

Serum ACE (17, 18) and sIL-2R (19, 20) are well-established activity markers in sarcoidosis. ACE, produced by activated monocytes, AMs, and epithelioid cells within granulomas (31, 32), rises in active disease, while sIL-2R reflects monocyte/macrophage activation and correlates with pulmonary function decline (20) and progression risk (33). However, both markers exhibit only modest sensitivity and specificity, and normal levels do not exclude sarcoidosis (34). The ATS/ERS/WASOG 2020 guidelines emphasize that these biomarkers should be used only as adjunctive tools for diagnosis and activity assessment (16). Within this context, direct morphological evaluation of AMs may provide complementary insights into disease activity.

This study has several limitations: (i) the controls comprised four young healthy male never-smokers and were not age- or sex-matched to the sarcoidosis cohort, which may introduce demographic variability in AM morphology. However, all samples were processed and quantified under identical staining conditions and the same digital planimetric workflow, and the magnitude of AM enlargement/activation observed in sarcoidosis is unlikely to be fully explained by demographic differences alone. In addition, to the best of our knowledge, there is no clear evidence that age or sex materially alters human AM morphology, and the AM sizes in our controls were comparable to those reported previously (13, 35). Although larger age−/sex-matched control cohorts would be ideal, obtaining BALF from healthy volunteers is ethically and practically challenging, which may limit the feasibility of such studies. (ii) The study had a retrospective, single-center design. (iii) The morphometric analysis relied on digital microscopy and semi-automated planimetry and may require further standardization for broader implementation. (iv) a focus on CA without fully characterizing ultrastructural subtypes (e.g., Burkhardt’s Types I–III) (7) or nuclear metrics (e.g., form factor) (12), and without addressing AM ontogeny (36); and (v) the absence of comparative data from other respiratory diseases (e.g., hypersensitivity pneumonitis or drug-induced pneumonitis). Nevertheless, our findings support a positive correlation between AM enlargement, morphological features, and disease activity.

Future studies should integrate multi-omics approaches to link morphological features of AMs with molecular profiles, thereby identifying the signaling pathways driving enlargement and activation. In addition, single-cell and lineage-tracing analyses will be needed to clarify the contributions of resident versus monocyte-derived subsets and to define the pathological roles of enlarged AMs in sarcoidosis.

In summary, digital morphometry showed that enlarged, vacuolated, rosette-forming AMs were more strongly associated with serum ACE and sIL-2R levels than the BALF CD4/CD8 ratio or radiological stage. Quantitative assessment of AM morphology may therefore provide a useful surrogate for pulmonary sarcoidosis activity. From a practical standpoint, BAL-based AM morphometry offers a direct cellular readout of macrophage activation, but its clinical utility is constrained by invasiveness and limited feasibility for repeated assessments. In contrast, HRCT is widely available and essential for structural evaluation (including fibrotic change) but is less specific for inflammatory activity, whereas FDG-PET can help assess active inflammation and guide management in selected cases, albeit with higher cost and limited availability. Accordingly, AM morphometry is best viewed as complementary to imaging—potentially most useful during diagnostic work-up when BAL is clinically indicated and in research/clinical trial settings with standardized protocols. To support broader implementation, efforts to minimize inter-institutional variability will be critical; future validation studies should include cross-site reproducibility exercises, such as processing aliquots of the same BAL sample at multiple centers using an identical protocol and/or having multiple institutions acquire images from the same slides and perform independent analyses.

Our findings support the concept that elevations in serum ACE and sIL-2R, together with AM enlargement and morphological alterations, represent interrelated manifestations of AM activation in sarcoidosis.

## Data Availability

The original contributions presented in the study are included in the article/Supplementary material, further inquiries can be directed to the corresponding author.

## Footnotes

1
https://www.r-project.org
