# Supplementary figures and images for: Enlarged, activated alveolar macrophages as quantitative surrogates of disease activity in pulmonary sarcoidosis
Source: Front Med (Lausanne). 2026 Jan 22;13:1739663. doi: 10.3389/fmed.2026.1739663 (PMC12872854; doi:10.3389/fmed.2026.1739663)

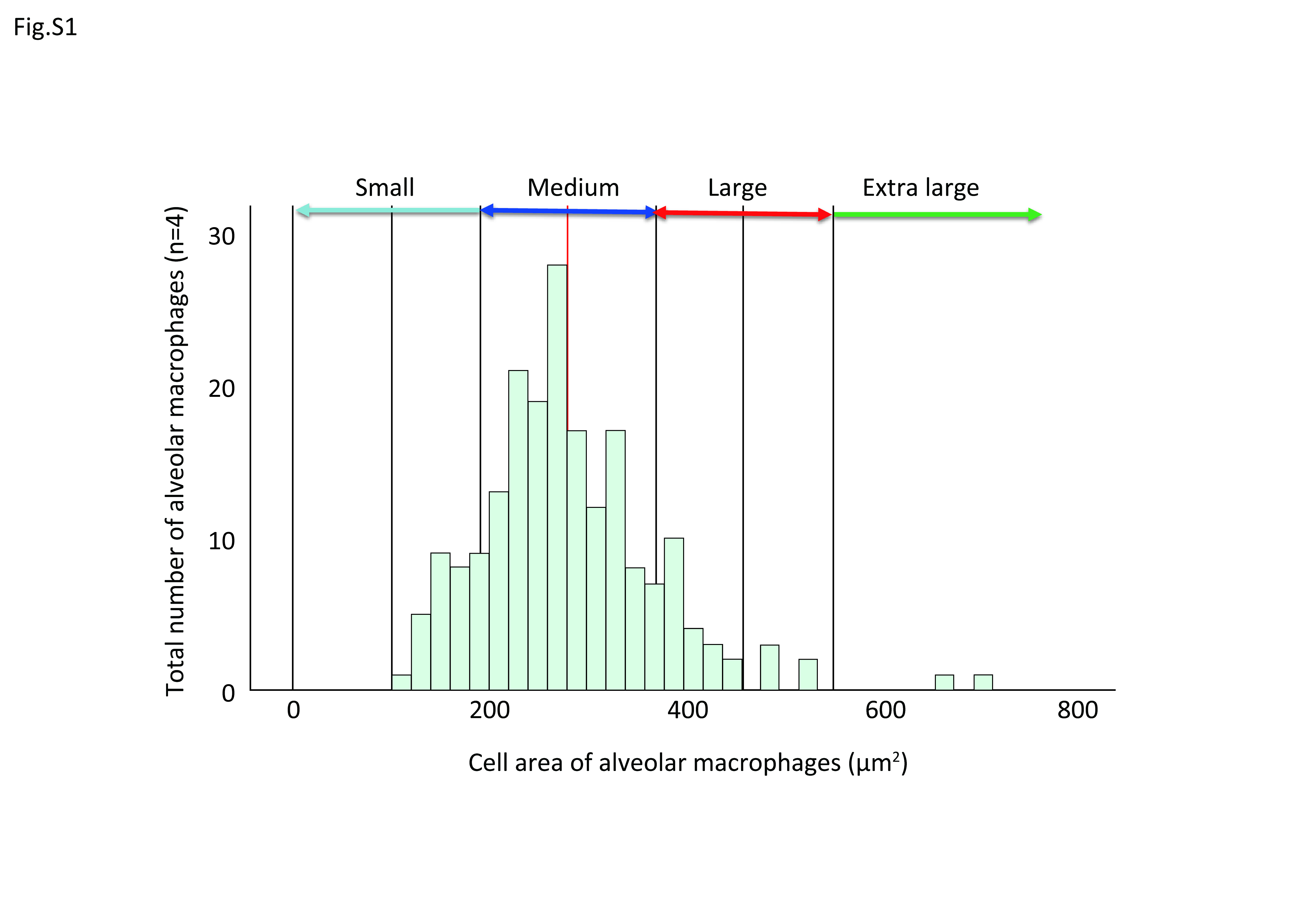

Supplement: Supplementary file 1 [file Image_1.TIF]

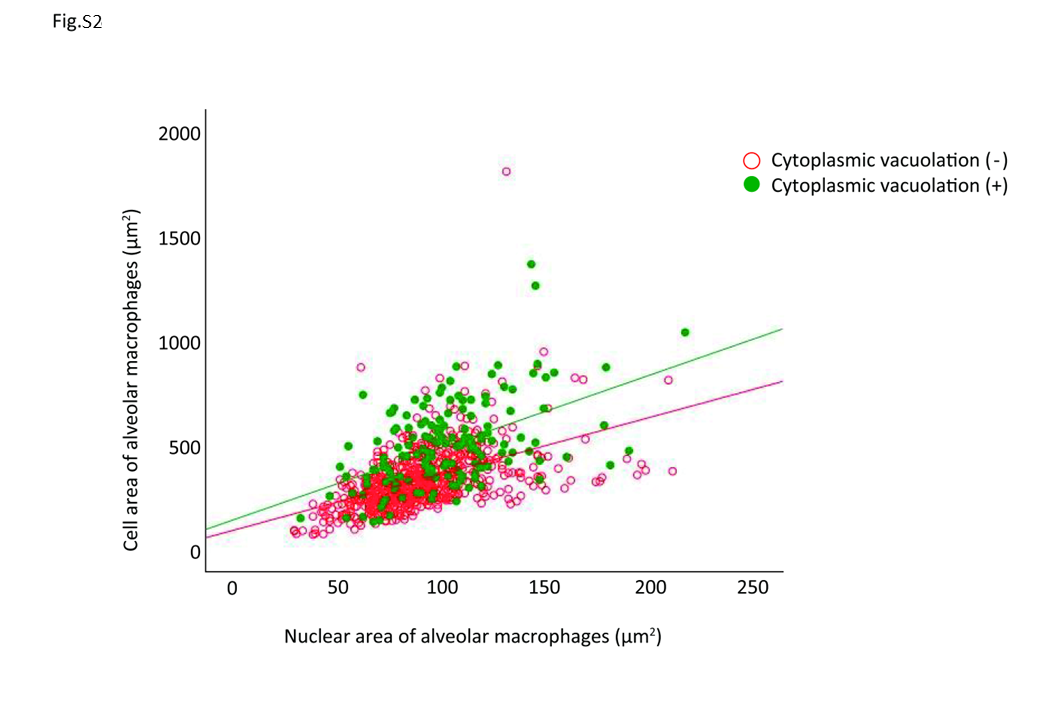

Supplement: Supplementary file 2 [file Image_2.TIF]

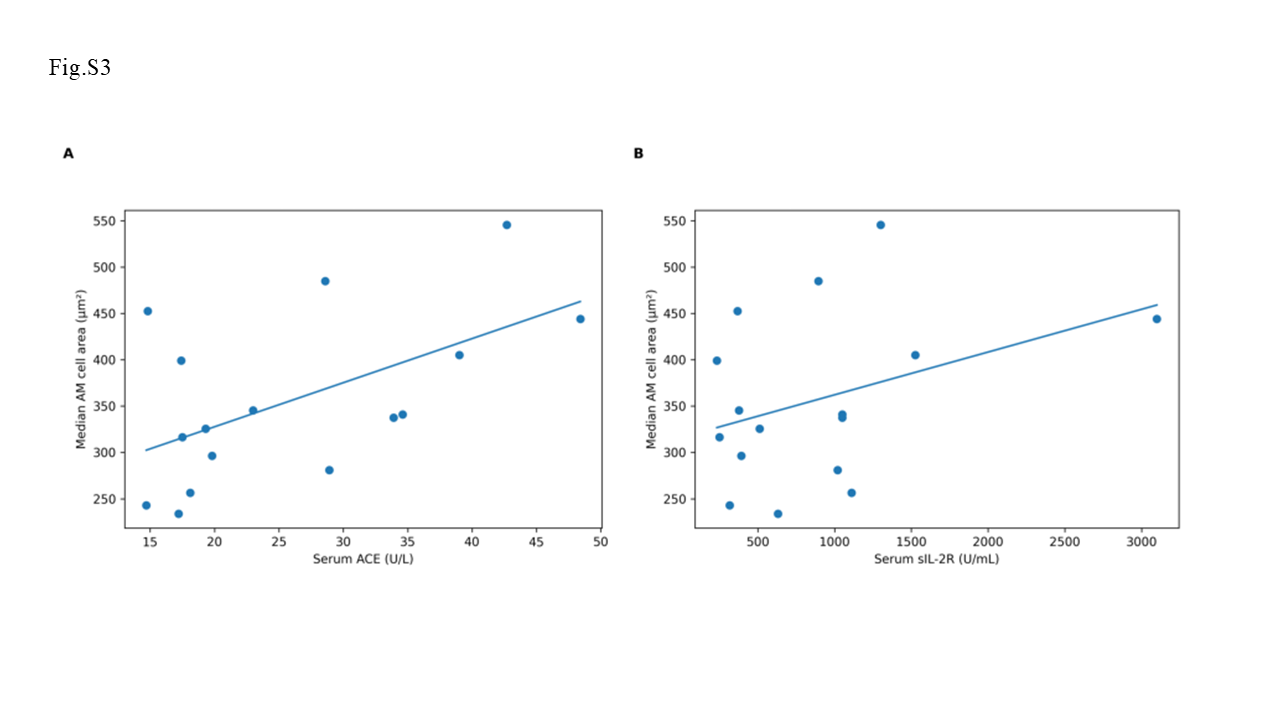

Supplement: Supplementary file 3 [file Image_3.TIF]
