# Supplementary material for: Enlarged, activated alveolar macrophages as quantitative surrogates of disease activity in pulmonary sarcoidosis
Source: Front Med (Lausanne). 2026 Jan 22;13:1739663. doi: 10.3389/fmed.2026.1739663 (PMC12872854; doi:10.3389/fmed.2026.1739663)
Supplement: Supplementary file 4 [file Table_1.docx]

Fig. S1

Distribution of cytoplasmic area (CA) in alveolar macrophages (AMs) from four healthy subjects (50 cells analyzed per case).

AMs are categorized into four groups based on CA measurements: small (< −1 SD, < 190.5 μm²), medium (within ±1 SD, 190.5–372.3 μm²), large (+1 SD to +3 SD, 372.3–554.1 μm²), and extra-large (> +3 SD, > 554.1 μm²).

Fig. S2

Stepwise Enlargement of Alveolar Macrophages According to Vacuolation and Rosette Formation

Fifty alveolar macrophages (AMs) are analyzed per case (n = 16). Vacuolation-positive AMs (mean ± SE, 500.3 ± 15.4 µm²) are significantly larger than vacuolation-negative AMs (337.1 ± 6.1 µm²; p < 0.001). Independently, AMs exhibiting rosette formation (474.7 ± 16.7 µm²; blue columns) have greater cell areas than those without rosette formation (349.4 ± 6.4 µm²; red columns; p < 0.001). Mean ± SE cell area increases progressively from left to right across the four columns—319 ± 6.0 µm², 423.5 ± 18.5 µm², 469 ± 17.4 µm², and 574 ± 29.5 µm²—demonstrating a stepwise enlargement of AMs.

Fig.S3

Case-level relationship between alveolar macrophage (AM) cell area and serum biomarkers (ACE and sIL-2R).

Scatter plots show the association between AM cell area (CA) and serum biomarkers measured at the case (patient) level: (A) CA versus serum angiotensin-converting enzyme (ACE) and (B) CA versus serum soluble interleukin-2 receptor (sIL-2R). For each case, 50 AMs were randomly selected, and the median CA of these 50 cells was used as the representative value for that case to avoid pseudo-replication arising from multiple cells per subject. Each point represents one case (n = 16). Correlations were assessed using Spearman’s rank correlation: CA (median) vs ACE, ρ = 0.503, p = 0.0471; CA (median) vs sIL-2R, ρ = 0.258, p = 0.336. A fitted line is shown for visual guidance.
